# Lung endothelial cells are sensitive to epsilon toxin from *Clostridium perfringens*

**DOI:** 10.1186/s13567-020-00748-2

**Published:** 2020-02-24

**Authors:** Jonatan Dorca-Arévalo, Eduard Dorca, Benjamín Torrejón-Escribano, Marta Blanch, Mireia Martín-Satué, Juan Blasi

**Affiliations:** 1 Department of Pathology and Experimental Therapeutics, Faculty of Medicine and Health Sciences, Campus of Bellvitge, University of Barcelona, Hospitalet de Llobregat, Barcelona, Spain; 2 Biomedical Research Institute of Bellvitge (IDIBELL), L’Hospitalet de Llobregat, Barcelona, Spain; 3 Institute of Neurosciences, University of Barcelona, 08035 Barcelona, Spain; 4 Pathology Service, Bellvitge University Hospital, L’Hospitalet de Llobregat, Barcelona, Spain; 5 Centres Científics i Tecnològics, Universitat de Barcelona, Campus Bellvitge, Barcelona, Spain; 6 Biomedical Research Institute of Bellvitge (IDIBELL), Oncobell Program, CIBERONC, L’Hospitalet de Llobregat, Barcelona, Spain

## Abstract

The pore-forming protein epsilon toxin (Etx) from *Clostridium perfringens* produces acute perivascular edema affecting several organs, especially the brain and lungs. Despite the toxin evident effect on microvasculature and endothelial cells, the underlying molecular and cellular mechanisms remain obscure. Moreover, no Etx-sensitive endothelial cell model has been identified to date. Here, we characterize the mouse lung endothelial cell line 1G11 as an Etx-sensitive cell line and compare it with the well-characterized Etx-sensitive Madin-Darby canine kidney epithelial cell line. Several experimental approaches, including morphological and cytotoxic assays, clearly demonstrate that the 1G11 cell line is highly sensitive to Etx and show the specific binding, oligomerization, and pore-forming activity of the toxin in these cells. Recently, the myelin and lymphocyte (MAL) protein has been postulated as a putative receptor for Etx. Here, we show the presence of *Mal* mRNA in the 1G11 cell line and the presence of the MAL protein in the endothelium of some mouse lung vessels, supporting the hypothesis that this protein is a key element in the Etx intoxication pathway. The existence of an Etx-sensitive cell line of endothelial origin would help shed light on the cellular and molecular mechanisms underlying Etx-induced edema and its consequences.

## Introduction

*Clostridium perfringens* types B and D produce severe and rapidly fatal enterotoxemia in ruminants, causing a lethality rate as high as 100% and substantial economic losses [1]. Both strains produce one of the most lethal clostridial toxins, the epsilon toxin (Etx). Etx is a protein synthesized as a very low-active molecule, the epsilon prototoxin (pEtx), by the bacteria present in the gut and becomes fully active after proteolytic cleavage of the C and N terminal residues [2, 3]. Once active, Etx alters intestinal permeability [4], enters the gut vasculature, and permeabilizes vascular endothelia [5], leading to various histological changes that include brain, lung, and heart edema in sheep and necrotizing colitis, pulmonary edema, and hydropericardium in goats [6, 7]. Following intravenous (i.v.) administration to mice, large amounts of radiolabeled Etx have been found in brain and kidneys with small amounts in heart, lungs, liver, and stomach [8]. Similar results have been observed in mice after i.v. administration of acute doses of Etx bound to green fluorescent protein (GFP-Etx), with Etx mainly being detected in kidneys, producing the death of epithelial cells from distal tubules and brain [9, 10]. It has also been demonstrated that Etx crosses the blood-brain barrier (BBB) [11], producing permeabilization [12–16] and endothelial cell damage in brain microvasculature [17]. The recombinant protein GFP-Etx has been successfully used as a tool to study Etx binding and toxic effects [9, 18]. Etx [19–21] and GFP-Etx mutants [22] have been tested by i.v. administration to mice. Mutants without a lethal effect (GFP-Etx-V56C/F118C and GFP-EtxH106P) did not cross the BBB [22], suggesting a direct correlation between the capacity of Etx to cross the BBB and lethality [23–26].

In addition, the effects of i.v. injection of Etx have been studied in a series of other animal species [27]. These studies have shown that Etx is required to induce most of the effects of natural intoxication, producing generalized edema in many tissues and organs, the most significant effects being acute pulmonary and cerebral edema [2, 28]. Moreover, the interstitial perivascular and alveolar edema produced in lungs of sheep and goats inoculated intraduodenally with *Clostridium perfringens* type D were attributable to the action of Etx [6]. Transmission electron microscopy has shown that the effect of Etx on vascular endothelia is associated with endothelial cell injury rather than intercellular junction disassembly [5, 15]. Thus, Etx has been directly implicated in vascular endothelium damage. Moreover, Adamson and collaborators perfused Etx in mesentery venular microvessels of rats and found increased vessel wall permeability with direct damage to the endothelium [5]. However, attempts to reproduce these effects in vitro with endothelial cell cultures from aortic segments have been unsuccessful [29]. There is currently no cellular model for in vitro studies, and previous research using endothelial cell lines from various animal species (including human umbilical endothelial cells) has not shown any effect of Etx [29], suggesting that only a selected set of endothelial cells are sensitive to Etx.

At the cellular level, most of the steps in the cytotoxic mechanism of Etx have been defined using the Madin–Darby canine kidney (MDCK) epithelial cell line: a canine renal tubular cell model that is highly sensitive to Etx. In this model, the toxin forms pores in the cell plasma membrane after binding and self-assembly forming oligomers [2, 11, 30]. Moreover, Etx shows a high structural homology to aerolysin, a pore-forming toxin (PFT), and consequently has been included in the aerolysin family of PFTs [31]. One of the main features of Etx is its high specificity to cellular models. Besides MDCK, very few cell lines are sensitive to the toxin, a characteristic that depends on the expression of a putative receptor, or receptors, on the cell surface, and the most promising candidate proteins are located in the detergent-resistant membrane domains or lipid rafts. It has been shown that the myelin and lymphocyte (MAL) protein, a lipid raft-associated protein [32] present in myelin and lymphocytes, is essential for Etx activity and is thereby considered a possible cell membrane receptor for Etx [33]. Moreover, Etx causes selective death of oligodendrocytes, the mature myelin-producing cells in the central nervous system [34], leading to demyelination [35], and this effect is dependent on MAL expression [34]. In addition, Etx produces cytotoxicity in T-lymphocytes, which also express the MAL protein [36]. Furthermore, the MAL protein has been identified in polarized epithelial cell lines, including the renal MDCK cell line [37, 38] and Fischer rat thyroid (FRT) cells [39], in which Etx is also cytotoxic [40]. The MAL protein has also been detected in many tissues such as myelin structures of the nervous system, distal and collecting tubules of the renal system, high endothelial venules of the lymph nodes, cortical thymocytes of the thymus, the epithelium of the bronchi and bronchioles of the lung, and type 2 pneumocytes [41].

Here we report the effects of Etx on an endothelial cell line, mouse lung endothelial 1G11 cells [42]. The proposed cytotoxic mechanism of action is similar to that observed in MDCK cells and follows the defined steps of binding, oligomerization, and pore formation in the cell membrane.

## Materials and methods

### Cloning, expression, and purification of pEtx, GFP-pEtx and GFP-pEtxH106P

pEtx wild type was produced as a fusion protein with (GFP-pEtx) or without GFP (pEtx), starting at amino acid A47 as described previously [11, 43] and lacking the N-terminal but not the C-terminal peptide, which was removed by trypsin digestion to produce active toxin when required.

Wild type DNA was cloned into the pGEX-4T-1 vector (GE Healthcare Life Sciences, Marlborough, MA, USA) containing the enhanced green fluorescent protein (EGFP) coding sequence to produce the corresponding recombinant fusion protein as previously described [11]. GFP-EtxH106P was generated as described previously [22], using the QuikChange multi-site-directed mutagenesis kit (Stratagene, San Diego, California, USA). Briefly, protein expression was induced overnight in the presence of 1 mM isopropyl beta-D-thiogalactopyranoside (IPTG) at room temperature (RT), in 250 mL of Luria-Bertani (LB) medium containing 50 µg/mL of ampicillin. Cells were pelleted and resuspended in 20 mM ice cold phosphate buffer (PB) pH 7.5 with 250 mM NaCl, sonicated, and centrifuged at 12 000 × *g* for 20 min at 4 °C. The resultant supernatant was incubated with Glutathione Sepharose^TM^ 4B beads (GE Healthcare Life Sciences) for 1 h at 4 °C. Finally, the recombinant protein was eluted by thrombin cleavage in PB containing 2.5 mM CaCl_2_.

### Activation of epsilon toxin by trypsin

Purified pEtx, GFP-pEtx and GFP-pEtxH106P were activated with trypsin to form activated Etx, GFP-Etx and GFP-EtxH106P [20]. Preparations containing pEtx, GFP-pEtx or GFP-pEtxH106P were incubated with trypsin-agarose beads (Sigma-Aldrich, St. Louis, MO, USA) at RT for 60 min, and then the trypsin-coated beads were removed by centrifugation. Protein concentration was determined by Bradford’s method [44] using bovine serum albumin (BSA) as standard. Conversion efficiency to Etx, GFP-EtxH106P and GFP-Etx was analyzed with SDS-PAGE and Coomassie blue staining.

### Immunohistochemical analysis of mouse samples

Male C57BL/6 J mice weighing approximately 20 g were housed in standard conditions in temperature-controlled, pathogen-free rooms with free access to standard pelleted food and tap water. The experiments were carried out in the animal research facility of the University of Barcelona (Bellvitge Campus) in animal preparation rooms equipped with the apparatus necessary for isoflurane anesthesia.

Mice were i.v. injected with GFP-Etx or GFP-EtxH106P at a final concentration of 2.5 μg/g with phosphate buffered saline (PBS)-1% BSA in a final volume of 50 µL, and control mice were only injected with PBS-1% BSA. Prior to the i.v. injections, the mice were anesthetized with isoflurane and the anesthesia was maintained with the same anesthetic until the end of the experiment.

All the animals injected with GFP-Etx died between 5 and 10 min after injection. No lethal effect was observed with either GFP-EtxH106P or control, and mice were sacrificed by cervical dislocation 10 min after injection. The mice were routinely processed for histopathological analysis, and each experimental procedure was performed three times in duplicate.

In order to perform immunohistochemical analysis of control, non-injected mice, the animals were anesthetized with intraperitoneal administration of ketamine (100 mg/kg) and xylazine (10 mg/kg) and then perfused by a gravity-fed system with 4% paraformaldehyde (PFA) via the vascular system for 20 min.

Lung sections from injected mice were mounted on SuperFrost^®^ Plus Microscope Slides (#631-0108, SuperFrost, VWR, Leuven, Belgium), and antigen retrieval was accomplished by subjecting deparaffinized sections to pressure-cooker unmasking (2100 Antigen retriever, BioVendor, Kassel, Germany) in 10 mM Tris and 0.5 mM EDTA at pH 9.0. Endogenous peroxidase activity was blocked with 10% methanol (v/v) and 2% H_2_O_2_ (v/v) in PBS for 30 min. The slides were pre-incubated for 1 h at RT with PBS containing 20% normal goat serum (NGS; Gibco, Paisley, UK), 0.2% Triton, and 0.2% gelatin (Merck, Darmstadt, Germany). Then, the sections were incubated overnight at 4 °C with the mouse monoclonal anti-MAL E1 antibody (sc-390687, Santa Cruz Biotechnology, Dallas, Texas, USA) or the rabbit polyclonal anti-pEtx [9] at 1:100 diluted in PBS containing 1% NGS, 0.2% gelatin, and 0.2% Triton. Samples were washed and incubated with the appropriate anti-mouse or anti-rabbit EnVision+ system- horseradish peroxidase (HRP) labeled polymer, and sections were developed by peroxidase reaction as explained in [45]. As a control, sections were treated identically but omitting the incubation with the primary antibody. Nuclei were counterstained with hematoxylin, and slides were then dehydrated and mounted with dibutylphthalate polystyrene xylene (DPX) mounting medium. Slides were examined in a Leica DMD108 digital micro-imaging system (Leica, Wetzlar, Germany).

### Cell lines

The MDCK cell line was purchased from (ATCC^®^ CCL-34^TM^, Manassas, Virginia, USA), the HeLa cell line was purchased from (ATCC^®^ CCL-2^TM^), the NIH/3T3 cell line was kindly provided by the laboratory of Dr Ricardo Pérez Tomás (Dept. Pathology and Experimental Therapeutics, Faculty of Medicine, Campus Bellvitge, University of Barcelona, Spain) and the murine lung endothelial 1G11 cell line was originally obtained by Dr Alberto Mantovani and Dr Annunciata Vecchi (Instituto Ricerche Farmacologiche Mario Negri, Milan, Italy).

MDCK and HeLa cells were maintained in Dulbecco’s modified essential medium (DMEM)-F12 medium (31330-038, Gibco) supplemented with 10% fetal bovine serum (FBS; Gibco\Invitrogen, Grand Island, NY, USA), and 50 U/mL penicillin/streptomycin (P0781, Sigma-Aldrich).

NIH/3T3 cells were maintained in DMEM medium (D6429, Sigma-Aldrich) supplemented with 10% FBS and 50 U/mL penicillin/streptomycin.

The 1G11 cells were maintained in DMEM-F12 medium supplemented with 20% FBS, 50 U/mL penicillin/streptomycin, 150 μg/mL endothelial cell growth supplement (356006, Corning, NY, USA), 100 μg/mL heparin (H3149, Sigma-Aldrich), 1% non-essential amino acids (M7145, Sigma-Aldrich), and 2 mM sodium pyruvate (11360-070, Gibco).

Cells were all grown at 37 °C in a humidified atmosphere of 5% CO_2_.

In all experiments, MDCK, HeLa and 1G11 cells were grown to confluence, but NIH/3T3 cells were grown up to 70% confluence according to the manufacturer’s instructions.

### Confocal microscopy

The cells were grown on coverslips and incubated for 30 min with 300 nM GFP-pEtx or GFP as a negative control, using the same conditions and culture medium. After three washes with PBS, the cells were fixed with 4% paraformaldehyde (PFA) for 12 min at RT. After another 3 washes with PBS, the samples were stained with TO-PRO-3 (1:1000 dilution, Molecular Probes, Invitrogen, Eugene, Oregon, USA) for 7 min, washed again, and mounted with the Fluoromount aqueous mounting medium (F4680, Sigma-Aldrich).

To study and compare the distribution of the cortical actin from Etx and pEtx treated cells, a time-course experiment at 15, 30, 60 and 120 min was performed incubating MDCK, NIH/3T3 and 1G11 cells with 50 nM Etx or pEtx. Next, the cells were fixed with 4% PFA for 12 min RT and washed thrice with PBS prior to incubation with CytoPainter Phalloidin-iFluor 555 reagent (ab176756, Abcam, Cambridge, UK) for 1 h at RT in order to stain the actin filaments (F-actin). The cells were washed thrice with PBS, the nuclei were stained with TO-PRO-3 and the cells were washed and mounted as explained above.

Coverslips were examined in a Leica TCS-SL spectral confocal microscope (CCiTUB, Bellvitge Campus, Biology Unit).

The viability of 1G11 and MDCK cells was recorded in a live imaging time-course experiment, exposing the cells to 50 nM of Etx, or pEtx as a negative control, during 120 min at 37 °C. Propidium iodide (PI, P4170, Sigma-Aldrich) was used in order to detect dead cells. Briefly, cells were grown in a chambered polymer coverslip for cell culture (80826, µ-Slide 8 well, ibidi, Gräfelfing, Germany) at a final volume of 300 µL/well. PI (red-fluorescent nuclear counterstain of dead cells) was added at 5 µL/mL and was incubated for 5 min in each condition previously to Etx or pEtx exposure. Photographs were taken every 77 s during 120 min in each condition, and a movie was generated overlapping all sections in each condition under a Zeiss LSM 880 Confocal Laser Scanning Microscope (CCiTUB, Bellvitge Campus, Biology Unit). Fluorescence images were processed with the ZEN 2.3 SP1 software (Zeiss).

### Cytotoxicity assay

MDCK, NIH/3T3 and 1G11 cells were grown in a 96-well plate, and the cytotoxic activity of wild type Etx was determined using the MTS Assay Kit (G3581, Promega, Madison, Wisconsin, USA). Briefly, the assay determines the capacity of living cells to reduce yellow tetrazolium MTS compound by viable cells to generate a colored formazan dye that is soluble in cell culture media. The formazan dye is quantified by measuring the absorbance at 490 nm.

The cells were exposed to increasing concentrations of GFP-Etx (0, 0.5, 1, 5, 10 and 50 nM) for 120 min at 37 °C. As a control, the cells were exposed to GFP-pEtx under the same conditions. Quadruplicates of each condition were performed.

The CT_50_ and LT_50_ for GFP-Etx were also determined. CT_50_ defines the concentration of Etx required to kill 50% of cells [19, 46], while LT_50_ (the median lethal time until death) is the mean time to kill 50% of cells [47].

LT_50_ was determined by exposing cells to 50 nM of GFP-Etx at different times (0, 15, 30, 60, 120, 180, and 240 min) at 37 °C. As a control, the cells were exposed to GFP-pEtx or GFP under the same conditions. Each condition was performed in quadruplicate.

### Luciferin-luciferase detection assay

The cells were plated onto a 96-well black polystyrene microplate (Merck) with a clear flat bottom and grown in 100 µL medium. Then, adenosine triphosphate (ATP) release from cells after GFP-Etx or GFP-pEtx exposure was measured using the luciferin-luciferase method. Luciferase extract lantern from Photinus pyralis (Sigma-Aldrich) was resuspended at 0.1 µg/µL and desalted in a 10 mL 10 DG column (Bio-Rad, Hercules, California, USA). d-Luciferin (Sigma-Aldrich) was diluted to a concentration of 0.7 µg/µL in ultrapure water and adjusted with NaOH to a final pH of 7.4. Then, a mixture of 5 µL of d-luciferin and 5 µL of luciferase was added to each cell well. The light emitted when ATP reacted with luciferin and luciferase was recorded using a FLUOstar OPTIMA Microplate Reader (BMG, Ortenberg, Germany) at the CCiTUB, Bellvitge Campus Biology Unit, University of Barcelona. Once the basal recording signal was stable, GFP-pEtx or GFP-Etx was added to each well to obtain the desired final concentration of 50 nM. When the bioluminescence peak returned to basal level, Triton X100 (final concentration of 0.2%) was added to evaluate the ATP content still present in the cells. Each condition was run in triplicate in three independent experiments.

### Oligomer complex formation

To analyze the formation of GFP-Etx protein complexes in MDCK and 1G11 cells, the cells were grown in 10-cm diameter plates and incubated with GFP-Etx for 15, 30, 60, and 120 min at 37 °C or with GFP-pEtx at 50 nM for 120 min in the same conditions. As a control, a plate was incubated only with cell medium for each cell type.

After incubation, the cells were washed once with PBS and then scraped off with 100 µL of ice cold RIPA buffer (25 mM Tris–HCl pH 7.4, 150 mM NaCl, 1% NP40, 0.1% SDS, 1% sodium deoxycholate) supplemented with 1:100 protease inhibitor cocktail (#P8340, Sigma-Aldrich). Harvested cells were lysed by passage through a 29-gauge needle (29G) and then sonicated on ice using an UP50H ultrasonic processor (Hielscher, Ultrasound Technology, Teltow, Germany) at 80% amplitude for ten cycles of 0.5 s each. Total cell lysates were quantified using the Pierce^TM^ BCA Protein Assay kit (Thermo Scientific, Massachusetts, USA), and 40 µg of each sample was electrophoresed in a precast polyacrylamide gel Mini-PROTEAN^®^ TGX^TM^ (#456-9033, Bio-Rad). Some wells were loaded with 0.5 ng of GFP-pEtx, GFP-Etx, or GFP recombinant protein as reference band. Later, a control assay was performed to compare the oligomer formation between MDCK and 1G11 and the non-sensitive NIH/3T3 cells. To do this, the cells were treated with 50 nM GFP-pEtx or GFP-Etx for 120 min at 37 °C and were lysed and quantified as explained above, and the samples were electrophoresed in a 10% polyacrylamide SDS-PAGE. Both the precast polyacrylamide gel and the 10% polyacrylamide SDS-PAGE were transferred to a nitrocellulose membrane Trans-Blot^®^ Turbo ^TM^ (#1704158, Bio-Rad) and analyzed by Western blot using anti-GFP rabbit polyclonal antibody (1:1000 dilution, [48]) followed by polyclonal swine anti-rabbit immunoglobulins/HRP (1:5000 dilution, #P0217, Dako, Glostrup, Denmark). To obtain the loading control signal, membranes were developed with anti-α-tubulin clone DM 1A (1:2000 dilution, #T9026, Sigma-Aldrich) followed by rabbit anti-mouse immunoglobulins/HRP (1:5000 dilution, #P0161, Dako). Membranes were developed with Luminata^TM^Crescendo western HRP substrate (Millipore, Massachusetts, USA), and the signal was detected using an Amersham Imager 600 (GE Healthcare Life Sciences).

### RNA isolation and semi-quantitative polymerase chain reaction (PCR)

Total RNA was extracted from kidneys and brain mouse samples using the RNeasy Lipid Tissue Mini Kit (#74804; Qiagen, Hilden, Germany), and the RNeasy Plus Mini Kit (#74134; Qiagen) was used in NIH/3T3 and 1G11 cells.

2.5 µg of RNA in each case was used for cDNA synthesis with the First Strand cDNA Synthesis kit (#K1612; Fermentas, Massachusetts, USA). Primers for mouse *Mal* detection were designed as:

*Ma*l-Forward: 5′-GCGAAGCTTATGGCTCCGGCAGCGGCTTCG-3′; and *Mal*-Reverse: 5′-GCGCTCGAGATGAAGACTTCCATCTGATTA-3′, containing *HindIII* and *XhoI* restriction sites (underlined), respectively.

PCR was developed as follows: 2 min at 95 °C followed by 40 cycles of 20 s at 95 °C, 10 s at 62 °C and 10 s at 70 °C. The product was analyzed in 2% agarose gel and RedSafe (21141, iNtRON Biotechnology, Burlington, USA) was used to visualize the DNA.

### Statistics

Cytotoxicity assay statistics were determined by nonlinear regression analysis using a two-way ANOVA with Tukey’s multiple comparison test. CT_50_ values for cytotoxicity tests were determined from MTS assay absorbance values using a nonlinear regression model (curvefit) based on the sigmoidal dose–response curve, log (inhibitor) vs. normalized response. CT_50_ was calculated with a 95% confidence interval (95% CI) indicated as 95% CI (lower–upper). LT_50_ values for the cytotoxicity test at 50 nM GFP-Etx were determined from MTS assay absorbance values over time, and a nonlinear regression (one-phase exponential decay) was performed and revealed a binding at time 0 (Y0 = 94.99%) and a nonspecific plateau (NS = 36.99%) considered as non-sensitive cells or background. In order to calculate the real LT_50_ another one-phase exponential decay was performed, but the Y0 and the NS were constricted to 94.99 and 36.99, respectively.

ATP released in Luciferin–Luciferase assay was calculated as the area under curve and statistics were determined by nonlinear regression analysis using a two-way ANOVA, followed by Sidak’s multiple comparisons test.

All statistics were calculated using GraphPad Prism version 7.00 for Windows (GraphPad Software, La Jolla, California, USA).

## Results

### Etx binds to the plasma membrane of 1G11 cells

**Figure 1:**
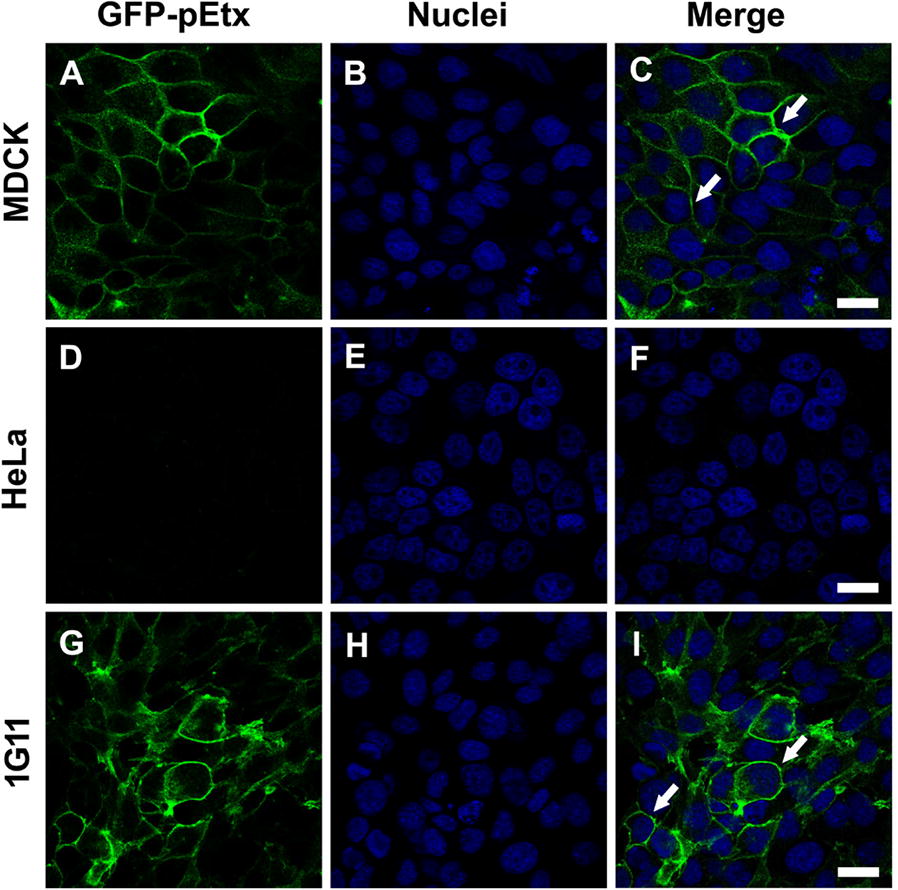
**Etx binds to the plasma membrane of 1G11 mouse endothelial cells**. Confocal microscopy images show the binding of GFP-Etx to the plasma membrane of MDCK cells, as a positive control (**A**–**C**) but not to HeLa cells, as a negative control (**D**–**F**). GFP-Etx also bound to the plasma membrane of the 1G11 cells (**G**–**I**), quite like MDCK cells. The cells were incubated with GFP-pEtx, and nuclei were stained with TO-PRO3 (Blue). Note GFP-pEtx binding to the plasma membrane of MDCK cells (arrows, **C**) and 1G11 cells (arrows, **I**). Scale bar 20 µm.

To study the effects of Etx on vascular endothelial cells, especially those related to the histopathological changes described in lung parenchyma of sheep and goats [49] and mice injected with GFP-Etx, we used the 1G11 cell line, a mouse lung capillary endothelial cell line. First, to determine Etx binding to 1G11 cells, we incubated the cells with the non-toxic form GFP-pEtx, which has a similar binding capacity as the toxic form [20], and the cells were analyzed by confocal microscopy. The images obtained revealed peripheral staining of GFP-pEtx in 1G11 cells (green, Figure 1G and arrows in I. See also the green mark in Additional file 1A and arrows in C) that were very similar to MDCK cells (green, Figure 1A and arrows in C), suggesting binding of the toxin to the plasma membrane. No GFP-pEtx binding was detected in the non-sensitive Etx HeLa cell line (Figures 1D–F) used as a negative control. Neither was binding detected in 1G11 cells incubated with GFP alone (see Additional files 1D–F), indicating that the binding detected was mediated by Etx and not by GFP.

### Etx is cytotoxic in 1G11 cells

**Figure 2:**
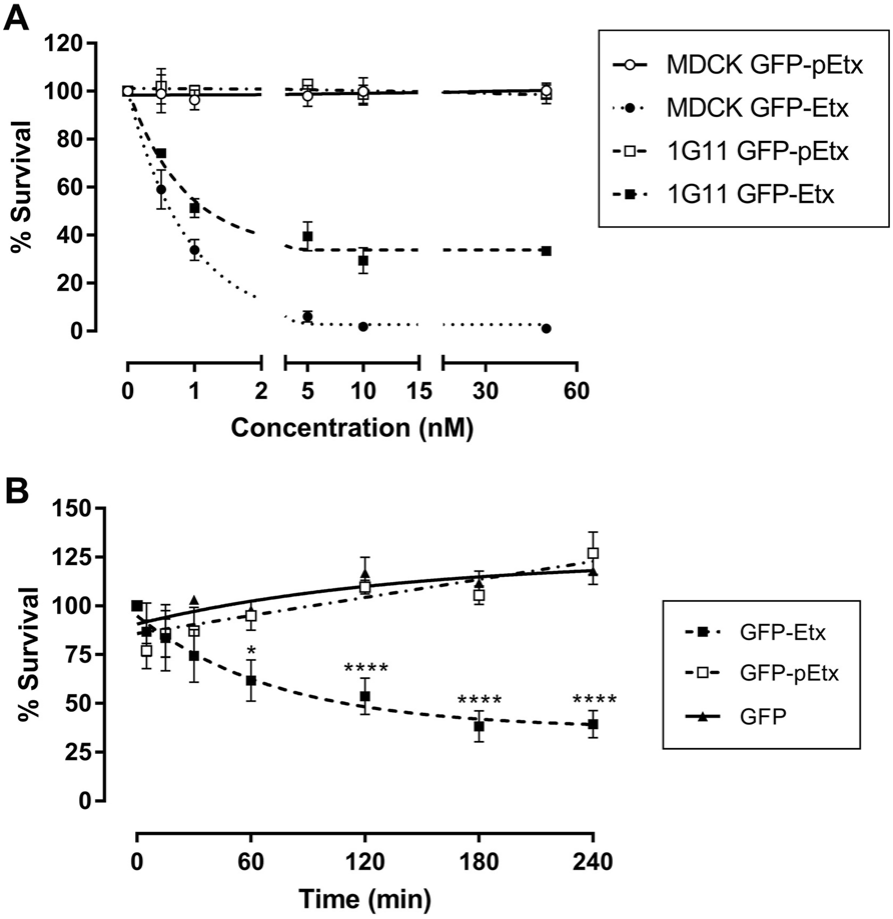
**Cytotoxic effect of GFP-Etx in 1G11 mouse endothelial cells**. 1G11 cells were exposed to increasing concentrations of GFP-Etx for 120 min at 37 °C, and the survival percentage was determined by the MTS assay (**A**). Around 70% of 1G11 dead cells were detected starting at 10 nM of GFP-Etx exposure compared to about 0% of dead cells detected with the non-toxic form of GFP-pEtx. The CT_50_ value for 1G11 cells was 2.17 nM (95% CI 1.05 to 4.58) and was 0.58 nM for MDCK cells (95% CI 0.48 to 0.69). Treatment with 50 nM of GFP-Etx on 1G11 cells for 240 min (**B**) revealed a LT_50_ of 50.84 min (95% CI 31.67 to 78.35) (*P = 0.02, ****P < 0.00001).

MTS cytotoxic assays were performed by incubating the cells with GFP-Etx for 120 min at different concentrations. The results revealed that MDCK cells were around four times more sensitive to GFP-Etx with a CT_50_ 0.58 nM (95% CI 0.48 to 0.69) than the 1G11 cells with a CT_50_ 2.17 nM (95% CI 1.05 to 4.58). No cytotoxic effect was detected for GFP-pEtx when incubated in 1G11 or in MDCK cells (Figure 2A).

To calculate the LT_50_ for GFP-Etx in the 1G11 cell line, the cells were exposed to 50 nM of GFP-Etx from 0 to 240 min, revealing a LT_50_ of 50.84 min (95% CI 31.67 to 78.35 min) (Figure 2B).

### Etx produces morphological changes and death of 1G11 cells

**Figure 3:**
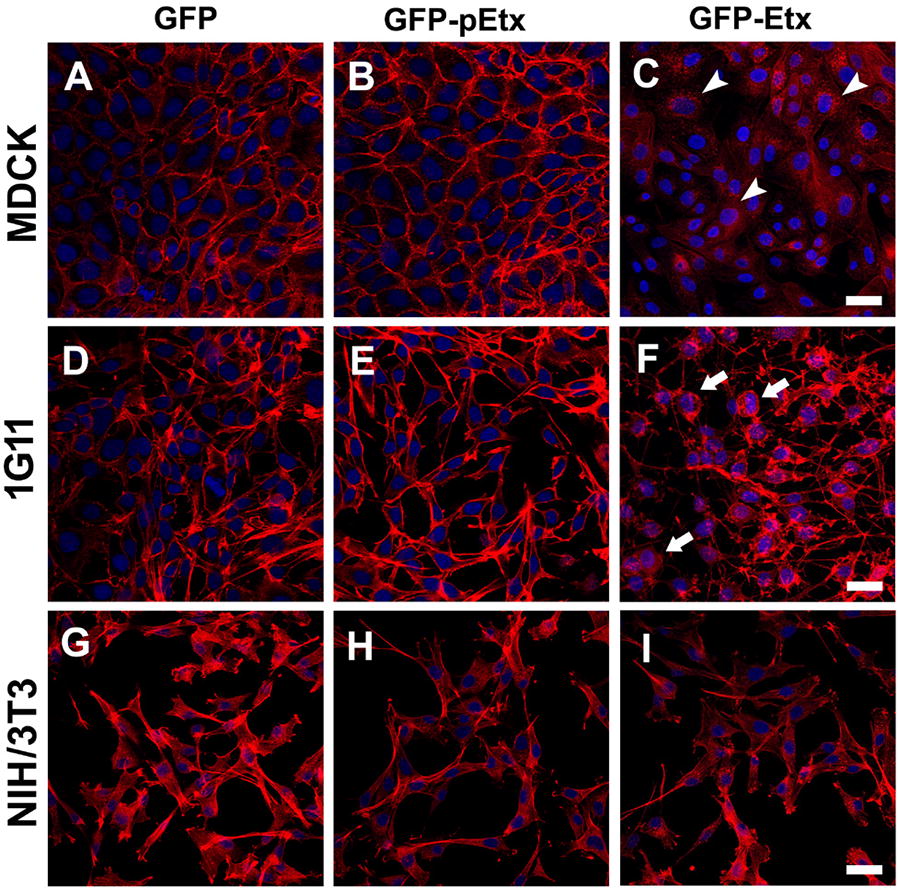
**Etx produces morphological changes in 1G11 mouse endothelial cells**. MDCK (**A**–**C**), 1G11 (**D**–**F**) and NIH/3T3 cells (**G**–**I**) were treated with 50 nM GFP (**A**, **D** and **G**), GFP-pEtx (**B**, **E** and **H**) or GFP-Etx (**C**, **F** and **I**) for 120 min at 37 °C. In MDCK and 1G11 cells, cortical actin was disorganized, changing its location from the plasma membrane to the cytosol (arrowheads in **C** and arrows in **F**, respectively). Note 1G11 cells with a more rounded morphology in GFP-Etx (**F**) compared to the elongated morphology in GFP or GFP-pEtx treatments (**D** and **E**, respectively). No changes were detected in NIH/3T3 cells. Bar 30 µm.

To visualize the effects of Etx on 1G11 cells by microscopic analyses, the cortical actin of cells treated with Etx at different periods of time was stained with phalloidin-Alexa 555. Etx produced disorganization of the cortical actin to a more cytosolic location, producing a consequent change in cell morphology (arrows, Figure 3F). This effect was not observed when cells were incubated for the same period (120 min) with GFP-pEtx of GFP alone (Figures 3E and D, respectively).

Similar results were observed in the highly sensitive MDCK cells (arrowheads, Figure 3C), and no changes were detected in cells treated with GFP-pEtx or GFP alone (Figures 3B, A, respectively). No changes were observed in the cortical actin or in the cell morphology of NIH/3T3 cells at 120 min of Etx exposure or with GFP-pEtx or GFP (Figure 3, panels I, H and G, respectively).

**Figure 4:**
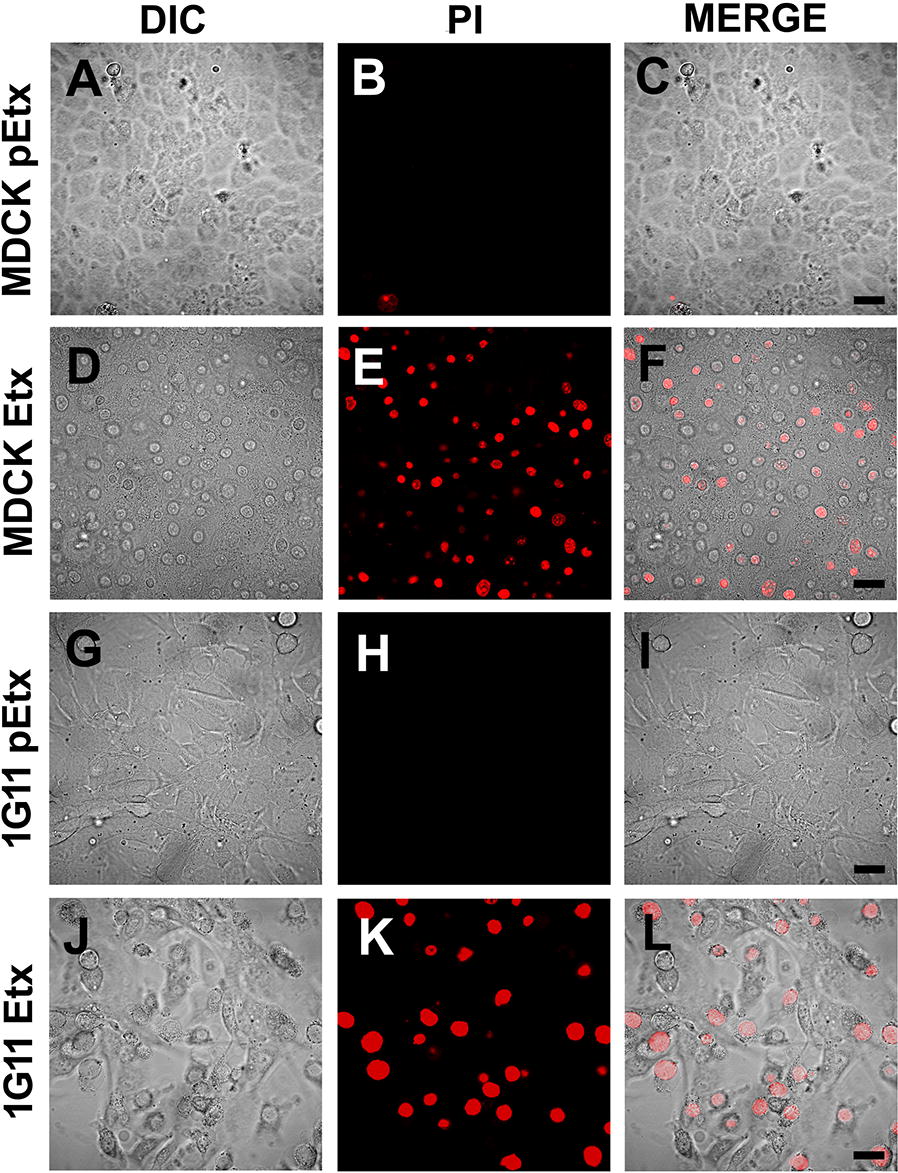
**Etx produces the death of 1G11 mouse endothelial cells**. MDCK cells (**A**–**F**) and 1G11 cells (**G**–**L**) were treated with pEtx (**A**–**C** and **G**–**I**) or Etx (**D**–**F** and **J**–**L**) for 120 min at 37 °C. Prior to pEtx or Etx treatment, the cells were incubated with propidium iodide (PI). Pictures correspond to 40 min of pEtx or Etx action in MDCK cells, and 90 min in 1G11 cells. Differential interference contrast (DIC) pictures were taken to see the change in cell morphology (**A**, **D**, **G** and **J**). Note a rounded morphology after Etx exposure compared to the elongated morphology in pEtx treatment in both MDCK and 1G11, and PI staining was also detected (red, **E** and **K**, respectively). Bar 25 µm.

To record cell death produced by Etx, live imaging in a time course assay was performed. Although death and morphological changes were detected in both MDCK and 1G11 cells, the effects on MDCK were faster than in 1G11 cells (see Additional files 2 and 3, respectively). No dead cells were detected with pEtx treatment in MDCK or 1G11 cells (see Additional files 4 and 5, respectively). Figure 4 shows selected images when the effects of Etx were evident and compared to pEtx exposition at the same interval time in each condition. No cell death or morphological changes were visualized in cells treated with pEtx compared to Etx (Figure 4).

### Etx induces ATP depletion in 1G11 cells

**Figure 5:**
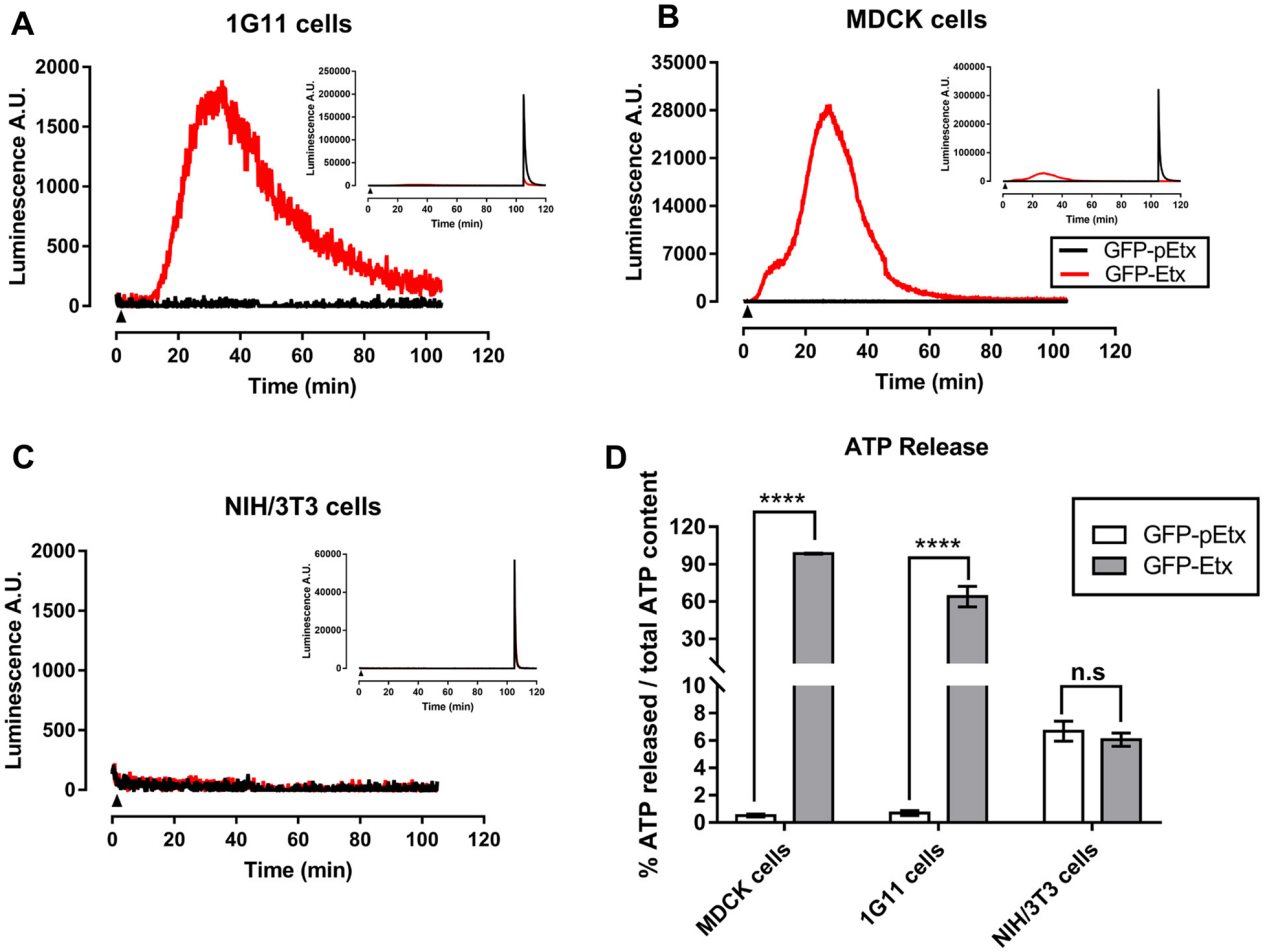
**Etx induces ATP release in 1G11 mouse endothelial cells**. ATP release was detected by GFP-Etx action in 1G11 cells (**A**) and in MDCK cells (**B**) but not in NIH/3T3 cells (**C**). The  % of ATP released with respect to the total ATP content was compared between GFP-Etx treated cells (gray columns, **D**) and GFP-pEtx treated cells (white columns, **D**). In **A**, **B** and **C**, cells were incubated with GFP-Etx (red line) or GFP-pEtx (black line) for 120 min. The cells were treated with Triton X-100 for the last 15 min to quantify total ATP content (inserts, **A**, **B** and **C**). GFP-Etx induced the release of 90% of the ATP content in MDCK cells; 60% in 1G11 cells and no ATP release was detected in NIH/3T3 cells, used as a negative control (**D**). Each condition was run in quadruplicate and in four independent experiments (****P < 0.00001), (n.s non-significant). Arrowhead marks the beginning of incubation with the GFP-Etx or GFP-pEtx.

Etx is a PFT that binds and oligomerizes in the plasma membrane of the host cell [30, 50]. The pore-forming capacity of Etx can be quantified by ATP release from cytosol or internal cell stores [36, 40]. To quantify the amount of ATP released, luciferin-luciferase assays were performed in 1G11 cells and were compared to MDCK (positive control) and NIH-3T3 cells (negative control). 1G11 cells revealed ATP release after GFP-Etx exposure (red line, Figure 5A), although the maximum peak value, around 2000 luminescence A.U, was about 15 times lower compared to the MDCK cells value which was around 28 000 luminescence A.U. (red line, Figure 5B). No ATP release was detected in the non-sensitive NIH/3T3 cell line treated with GFP-Etx (red line, Figure 5C) or in all the cell lines treated with GFP-pEtx (black line, Figures 5A–C). At the end of the experiment, all the ATP was virtually released by GFP-Etx in MDCK cells and no residual ATP could be measured after cell permeabilization with Triton X-100 (red line in the insert, Figure 5B). In 1G11 cells, almost all the ATP was released by GFP-Etx, and very little residual ATP was measured after cell permeabilization (red line in the insert, Figure 5A). However, all the ATP content was detected in NIH/3T3 cells after cell permeabilization (red line in the insert, Figure 5C). The area under curve revealed around 90% and 60% of ATP released from total ATP content in MDCK and 1G11 cells, respectively (Figure 5D).

### GFP-Etx oligomerizes in 1G11 cells

**Figure 6:**
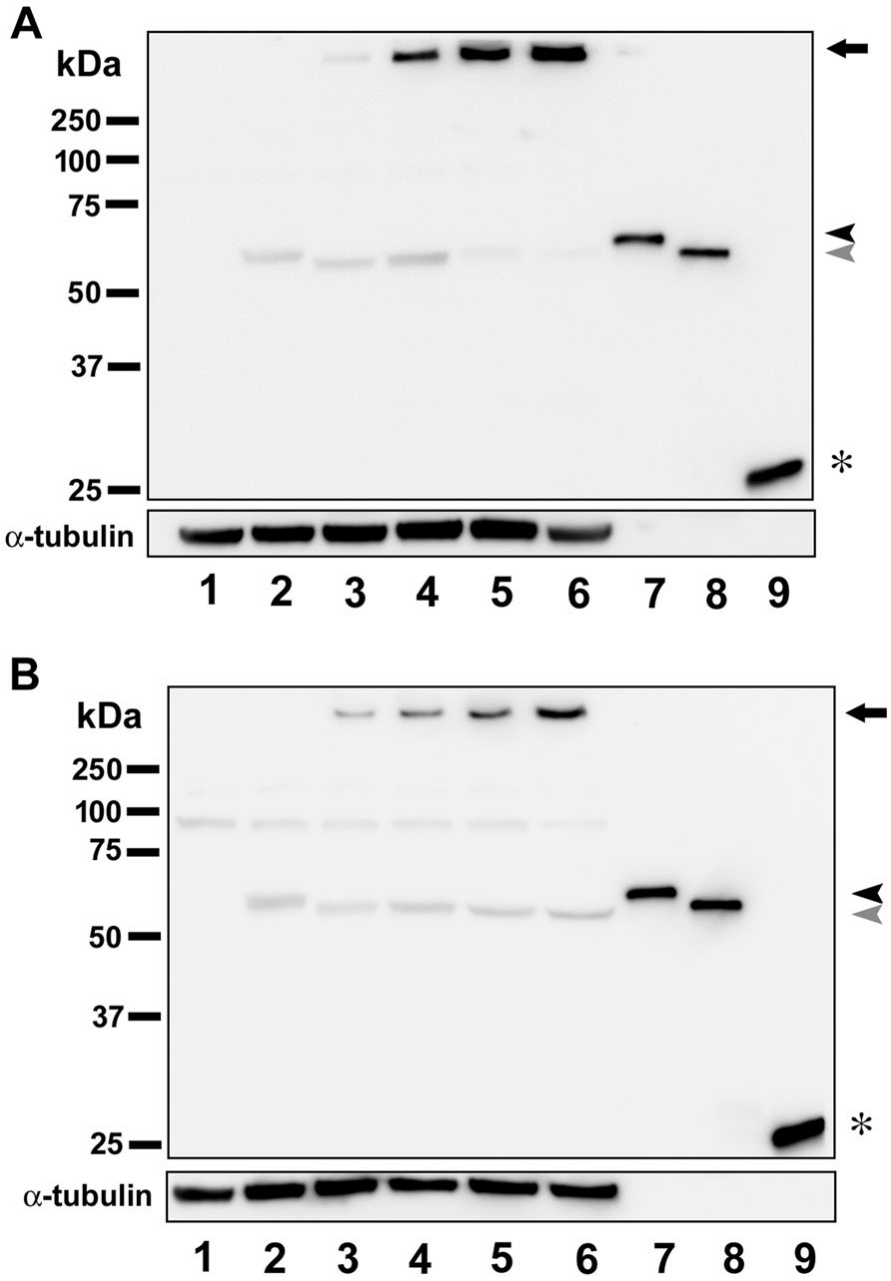
**Etx oligomerizes on 1G11 mouse endothelial cells**. MDCK (**A**) and 1G11 (**B**) cells were treated with 50 nM of GFP-pEtx or GFP-Etx for different incubation times (lane 1: non-treated cells; lane 2: cells treated with GFP-pEtx for 120 min exposure at 37 °C; lanes 3 to 6: cells treated with GFP-Etx for 15, 30, 60, and 120 min exposure at 37 °C, respectively; lane 7: GFP-pEtx recombinant protein; lane 8: GFP-Etx recombinant protein; and lane 9: GFP recombinant protein). Note the oligomer complex formation above 250 kDa (arrows, **A** and **B**) in both MDCK and 1G11 cells. See the GFP-pEtx around 60 kDa (black arrowhead, **A** and **B**), the GFP-Etx around 56 kDa (gray arrowhead, **A** and **B**), and the GFP around 27 kDa (asterisk, **A** and **B**). α-tubulin was used as a loading control.

To visualize Etx oligomer complex formation, 1G11 cells were incubated with 50 nM GFP-Etx for 15, 30, 60 and 120 min, and the results were compared to MDCK cells under the same conditions. As a negative control, cells were incubated, under the same conditions, with GFP-pEtx for 120 min (which does not form oligomers) or in the absence of the toxin. Western blot assays revealed similar oligomer complex formation (> 250 kDa) in MDCK cells (arrow, lanes 3 to 6, Figure 6A) as in 1G11 cells (arrow, lanes 3 to 6, Figure 6B). The monomeric form of GFP-pEtx, around 60 kDa, was detected in both MDCK and 1G11 cells incubated with GFP-pEtx (black arrowhead, lane 2, Figures 6A, B, respectively), confirming that GFP-pEtx binds to cells as a monomer but is ineffective in oligomer complex formation. No oligomer complex formation was detected in NIH/3T3 cells even at 120 min of incubation with GFP-Etx (see Additional file 6).

### Expression of MAL protein in endothelial cells from mouse lung

**Figure 7:**
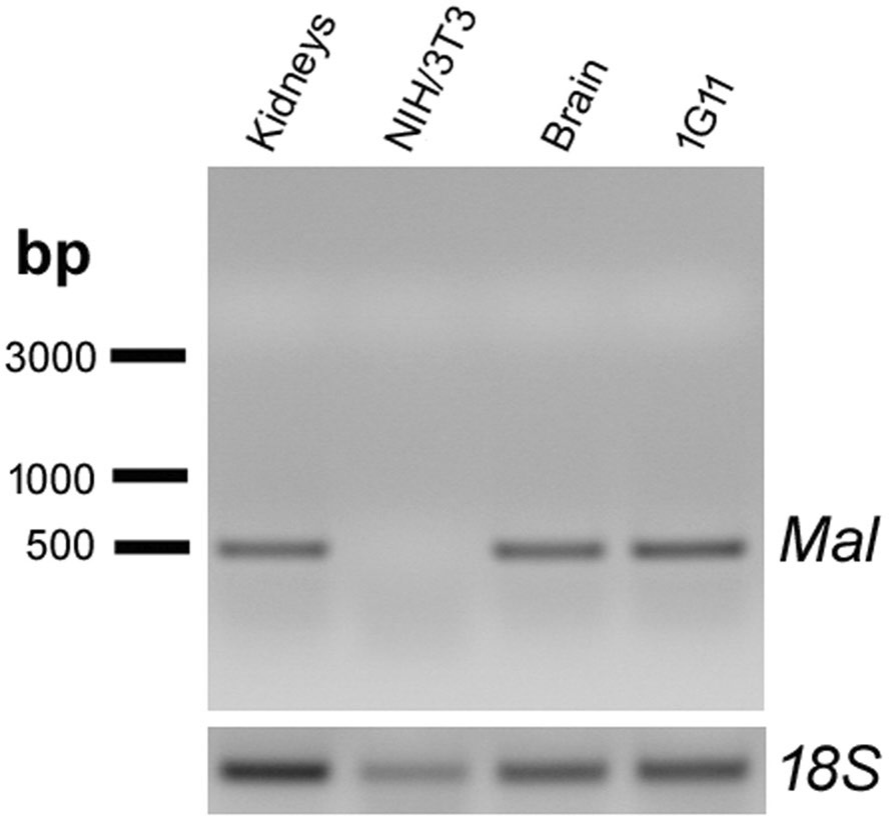
**The 1G11 mouse endothelial cell line expresses*Mal*RNA**. Reverse transcription-PCR detection of endogenous mouse *Mal* mRNA was extracted from kidneys and brain from mouse tissue (as a positive control) and from NIH/3T3 cells (as a negative control) and was compared to 1G11 cells. Samples were electrophoresed in 2% agarose gel. A band corresponding to *Mal* RNA weight was detected at around 500 bp in 1G11 cells and in brain and kidneys but not in NIH/3T3 cells. The 18S rRNA was used as a control. The DNA molecular weight marker is represented on the left (bp).

To investigate the hypothesis of the MAL protein acting as a putative receptor for Etx [33], we first evaluated the expression of *Mal* RNA in 1G11 cells by performing a RT-PCR assay. We found a single DNA electrophoretic band of around 500 bp in 1G11 cells and in brain and kidneys tissue as a positive control. No band was detected in NIH/3T3 cells used as a negative control (Figure 7).

**Figure 8:**
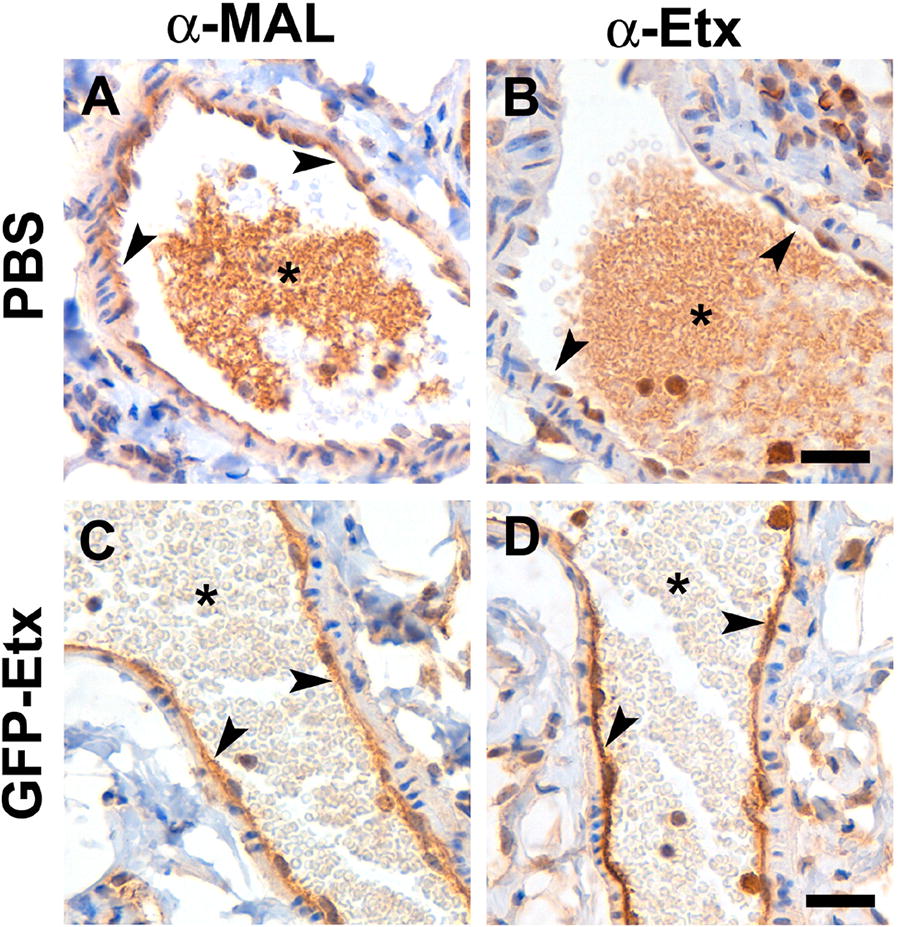
**Endothelial cells from mice lungs express the MAL protein**. Immunohistochemistry assays against the MAL protein (**A** and **C**) or Etx (**B** and **D**) on lung sections from mice injected with PBS (**A**, **B**) or GFP-Etx (**C**, **D**). MAL protein expression in the endothelial cells of some vessels was present in both PBS- and GFP-Etx-injected mice (brown, arrowheads in **A** and **C**, respectively). Etx was present in GFP-Etx-injected mice (brown, arrowhead in **D**) but not in the PBS control mice (arrowheads in **B**). Note unspecific staining from blood cells inside vessels in all conditions (brown, asterisk in **A**–**D**). Nuclei were stained with hematoxylin. Scale bar 25 µm.

The presence of the MAL protein and Etx in lung endothelial cells was detected by immunohistochemistry assays on lung sections from GFP-Etx- and PBS-injected mice incubated with α-MAL or α-Etx. While the MAL protein was present in the endothelium of some vessels in both, PBS- and GFP-Etx-injected mice (arrowheads, Figures 8A, C, respectively), Etx was only detected in the endothelium of GFP-Etx-injected mice (arrowheads, Figure 8D) but not in that of the PBS-injected mice (arrowheads, Figure 8B). Moreover, in control sections in which the primary antibody was omitted, no label was observed in the endothelium (arrowheads, Additional file 7).

In some samples, blood cells were labeled as a background (asterisks, Figure 8). In attempts to improve the background, a non-injected mouse was perfused with 4% PFA, and lung sections were fixed and processed. The MAL protein was detected in the endothelium of some vessels (arrows, Additional file 8D) but was not present in the endothelium of control samples (arrows, Additional file 8B) in which the primary antibody was omitted. Moreover, although there may be variations between species, the MAL protein has been detected in type 2 pneumocytes and in the bronchial epithelial cells [41], according to our results (arrowheads, Additional file 8D) and (asterisks, Additional file 8C), respectively.

## Discussion

In the present study, we used the 1G11 mouse endothelial cell line to further study the effect of Etx on endothelial cells. The results obtained, are relevant as this toxin produces edema in certain organs, including lungs and brain, as a major histological lesion [28]. The incubation of 1G11 cells with GFP-Etx revealed its binding to the plasma membrane followed by disorganization of the cortical actin, with a change in cell morphology showing the actin location to be more cytosolic instead of being distributed under the plasma membrane. These results were supported by Etx-induced cytotoxicity and cell death detected by either confocal microscopy, with an increase of PI staining of dead cells, or by MTS assay.

Etx effects in MDCK cells appeared faster than in 1G11 cells, possibly related to a difference in sensitivity to Etx in these different cell types. MTS assays revealed a CT_50_ of 2.17 nM, being around 4 times less sensitive for 1G11 cells compared to the CT_50_ of 0.58 nM in MDCK cells, and this value is similar to that of previous studies [36]. Moreover, the median lethal time (LT_50_) in 1G11 cells was 50.84 min, which is the optimal incubation time to detect 50% of cell death. Although 60 min of exposure in 1G11 cells was sufficient to produce statistical differences with respect to GFP-pEtx or GFP alone, we decided to perform viability assays for 120 min at 37 °C which would correspond around 2.5 times the LT_50_ for GFP-Etx in 1G11 cells. The difference between MDCK and 1G11 cells in GFP-Etx action may be due to the presence of different amounts of the Etx receptor among cell types or different efficacy in Etx oligomerization and pore formation. In support of this hypothesis, Western blot analysis revealed oligomer complex formation (> 250 kDa) both in MDCK and 1G11 cells exposed to GFP-Etx, although a thinner band was detected in 1G11 compared to MDCK cells, while no band was observed in MDCK and 1G11 cells following GFP-pEtx treatment. These results also suggest that the efficacy of oligomer complex formation and Etx sensitivity were lower in the 1G11 than in MDCK cell line. Consequently, under the same conditions, the luciferin-luciferase assay showed a luminescence peak value in both MDCK (28 000 A.U.) and 1G11cells (2000 A.U.) related to ATP release, but it was not detected in the non-sensitive NIH/3T3 cell line. In MDCK cells the peak value was 14 times higher than in 1G11 cells, which could be explained by a different ability to form Etx complexes due to a different amount of Etx receptor expression in each cell type or different mitochondrial population among cell lines with less ATP content in 1G11 cells compared to MDCK cells. Furthermore, the sensitivity to Etx is different in each cell type, becoming around 30–40% of non-sensitive in 1G11 cells compared to the less than 10% in MDCK cells, which may be attributable to different stages of cell physiology with poor Etx receptor expression.

The MAL protein has been implicated in specific Etx binding and cytotoxic activity, suggesting that MAL is a putative receptor for Etx [33, 34]. Moreover, MDCK cells express the MAL protein [32, 39], which is also expressed in other cells as MOLT4 or Jurkat [36] and FRT cells [40, 51], which are also sensitive to Etx. Here, we show Etx activity for the first time in an endothelial cell line in which *Mal* RNA is present, supporting the hypothesis of MAL as a key element of Etx action. Although we tested various MAL antibodies, none proved suitable to recognize the endogenous MAL protein in 1G11 or MDCK cells by confocal microscopy (data not shown). This may be due to low endogenous protein expression or failure of the antibodies tested to recognize the MAL protein from different species in culture cells. However, immunohistochemistry assays of mouse lung sections detected the MAL protein in the endothelium after antigen retrieval treatment. This label coincides with Etx detection in the endothelium of lung sections from GFP-Etx-injected mice, supporting the hypothesis of MAL as the putative receptor for Etx. MAL protein was also detected in the epithelium of bronchioles and in type 2 pneumocytes from mouse, coinciding with previous results obtained in human [41], although the effect of Etx on these cells remains unknown. However, MAL protein was not reported in human lung endothelium probably due to protein expression variability among species.

In conclusion, Etx binds, oligomerizes and produces a cytotoxic effect on the endothelial cell line 1G11 from mouse lung in a similar way as the well-characterized MDCK cell line. Accordingly, 1G11 cells become a new model to study the cellular and molecular mechanisms of Etx in the endothelium. These results suggest a cytotoxic effect of Etx on lung endothelial cells that would be responsible for the histopathological changes observed in Etx intoxicated animals. In addition, the MAL protein has been postulated to be the putative receptor for Etx; in fact, we have demonstrated the presence of *Mal* RNA expression in 1G11 cells, and MAL protein expression in the endothelium of some vessels from mouse lung, reinforcing the hypothesis of the MAL protein as the Etx receptor, or at least, that the presence of the MAL protein is crucial for the Etx binding and cytotoxic activity.

## Supplementary information

**Additional file 1. GFP-pEtx, but not GFP alone, binds to 1G11 mouse endothelial cells.** Confocal microscopy images show the binding of GFP-pEtx but not GFP alone, to the plasma membrane of 1G11 cells. The cells were incubated with GFP-pEtx (A–C) or GFP alone (D–F), and the nuclei were stained with TO-PRO3 (Blue, B, C, E and F). Note GFP-pEtx binding to the plasma membrane of 1G11 cells (green, in A and arrows in C) which was not detected in incubations with GFP alone (D and F). Scale bar 25 µm.

**Additional file 2. Etx produces MDCK cell death.** MDCK cells were pre-incubated with propidium iodide (PI) prior to treating the cells with Etx for 120 min at 37 °C. Differential interference contrast shows the cell morphology (grey), and PI was used to detect the nuclei of dead cells (red). Most of the cells were affected by Etx. Scale bar 20 µm.

**Additional file 3. Etx produces 1G11 mouse endothelial cell death.** 1G11 cells were pre-incubated with propidium iodide (PI) prior to treating the cells with Etx for 120 min at 37 °C. Differential interference contrast shows the cell morphology (grey), and PI was used to detect the nuclei of dead cells (red). Most of the cells were affected by Etx. Scale bar 20 µm.

**Additional file 4. pEtx does not produce MDCK cell death.** MDCK cells were pre-incubated with propidium iodide (PI) prior to treating the cells with pEtx for 120 min at 37 °C. Differential interference contrast shows the cell morphology (grey), and PI was used to detect the nuclei of dead cells (red). No MDCK cells were affected by pEtx. Scale bar 20 µm.

**Additional file 5. pEtx does not produce 1G11 mouse endothelial cell death.** 1G11 cells were pre-incubated with propidium iodide (PI) prior to treating the cells with pEtx for 120 min at 37 °C. Differential interference contrast shows the cell morphology (grey), and PI was used to detect the nuclei of dead cells (red). No 1G11 cells were affected by pEtx. Scale bar 20 µm.

**Additional file 6. Etx does not oligomerize in NIH/3T3 non-sensitive cells.** MDCK, 1G11 and NIH/3T3 cells were treated with 50 nM of GFP-pEtx (lane 1, 3 and 5, respectively) or GFP-Etx (lane 2, 4 and 6, respectively) for 120 min at 37 °C. Oligomer complex formation is detected above 250 kDa (arrow) in both MDCK and 1G11 cells but not in the NIH/3T3 cells. α-tubulin was used as a loading control. Note less intensity in 1G11 complex compared to MDCK cells complex.

**Additional file 7. Secondary-HRP antibodies did not bind to the endothelium from lung sections of injected mice.** Immunohistochemistry assays of lung sections from mice injected with PBS (A and B) or GFP-Etx (C and D) were revealed with anti-mouse EnVision+ system-HRP (A and C) or anti-rabbit EnVision + system-HRP (B and D) as a secondary antibody. Incubations were developed as explained in the Materials and Methods sections but omitting the primary antibody. No binding was detected in the endothelium of any condition (arrowheads), with only a little background of some blood cells from lungs sections being revealed with the secondary antibody (brown, A and C). Nuclei were stained with hematoxylin. Scale bar 25 µm.

**Additional file 8. Anti-MAL staining on mouse lungs.** Immunohistochemistry assays on lung sections from perfused mouse revealed MAL protein expression in endothelial cells of some vessels. Lung sections were incubated with the MAL antibody (C and D) or by omitting the primary antibody (A and B). All the sections were incubated with anti-mouse EnVision+ system-HRP and developed as explained in the Materials and Methods section. Note MAL protein expression in the endothelium (brown, arrows in D). However, it was not detected in the control conditions omitting the incubation with the primary antibody (arrows in B). MAL was also expressed in bronchial epithelial cells (brown, asterisk in C) and in type 2 pneumocytes (brown, arrowhead in D). B and D are magnifications from A and C images, respectively. Scale bars correspond to 50 µm in A and C, and 20 µm in B and D.

## Data Availability

The datasets supporting the conclusions of this article are included within the article.

## Abbreviations

ATP: adenosine triphosphate
A.U: arbitrary units
BBB: blood–brain barrier
bp: base pair
BSA: bovine serum albumin
CI: confidence interval
CT_50_: lethal dose 50
DAB: 3,3′-Diaminobenzidine
DIC: differential interface contrast
DMEM: Dulbecco’s modified essential medium
DPX: dibutylphthalate polystyrene xylene
EGFP: enhanced green fluorescent protein
Etx: epsilon toxin
GFP: green fluorescent protein
FRT: Fischer rat thyroid cells
FBS: fetal bovine serum
HRP: horseradish peroxidase
i.v.: intravenous
IPTG: isopropyl beta-d-thiogalactopyranoside
LB: Luria–Bertani
LT_50_: lethal time 50
MAL: myelin and lymphocyte protein
MDCK: Madin-Darby canine kidney cells
MTS: [3-(4,5-dimethylthiazol-2-yl)-5-(3-carboxymethoxyphenyl)-2-(4-sulfophenyl)-2H-tetrazolium]
NGS: normal goat serum
PB: phosphate buffer
PBS: phosphate buffered saline
pEtx: epsilon prototoxin
PFA: paraformaldehyde
PFT: pore-forming toxin
PI: propidium iodide
RT: room temperature
