# Supplementary figures and images for: Lung endothelial cells are sensitive to epsilon toxin from Clostridium perfringens
Source: Vet Res. 2020 Feb 24;51:27. doi: 10.1186/s13567-020-00748-2 (PMC7041264; doi:10.1186/s13567-020-00748-2)

GFP-pEtX

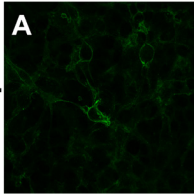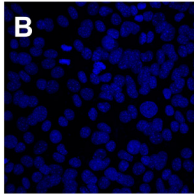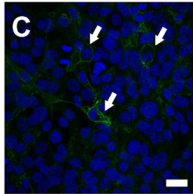

GFP

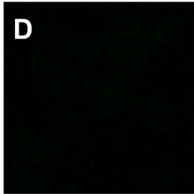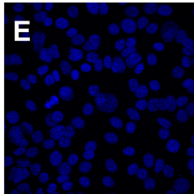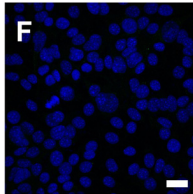

Supplement: Supplementary file 1 — Additional file 1. GFP-pEtx, but not GFP alone, binds to 1G11 mouse endothelial cells. Confocal microscopy images show the binding of GFP-pEtx but not GFP alone, to the plasma membrane of 1G11 cells. The cells were incubated with GFP-pEtx (A–C) or GFP alone (D–F), and the nuclei were stained with TO-PRO3 (Blue, B, C, E and F). Note GFP-pEtx binding to the plasma membrane of 1G11 cells (green, in A and arrows in C) which was not detected in incubations with GFP alone (D and F). Scale bar 25 µm. [file 13567_2020_748_MOESM1_ESM.pdf]

**MDCK**

**1G11**

**NIH-3T3**

**kDa**

**250** —

**150** —

**100** —

**$\alpha$ -tubulin**

**1**

**2**

**3**

**4**

**5**

**6**

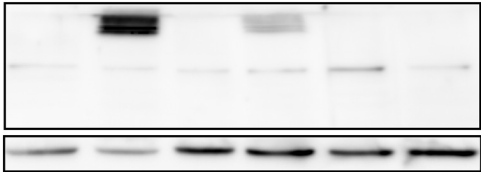

Supplement: Supplementary file 6 — Additional file 6. Etx does not oligomerize in NIH/3T3 non-sensitive cells. MDCK, 1G11 and NIH/3T3 cells were treated with 50 nM of GFP-pEtx (lane 1, 3 and 5, respectively) or GFP-Etx (lane 2, 4 and 6, respectively) for 120 min at 37 °C. Oligomer complex formation is detected above 250 kDa (arrow) in both MDCK and 1G11 cells but not in the NIH/3T3 cells. α-tubulin was used as a loading control. Note less intensity in 1G11 complex compared to MDCK cells complex. [file 13567_2020_748_MOESM6_ESM.pdf]

**$\alpha$ -mouse**

**$\alpha$ -rabbit**

**A**

**B**

**PBS**

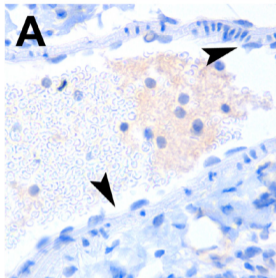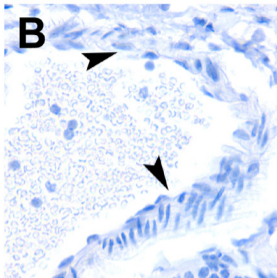

**C**

**D**

**GFP-Etx**

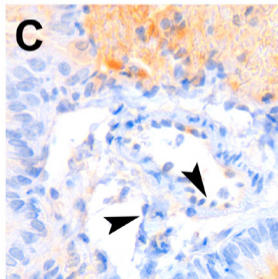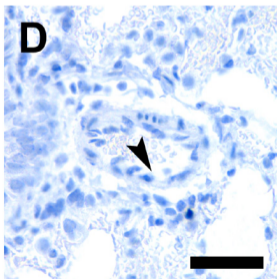

Supplement: Supplementary file 7 — Additional file 7. Secondary-HRP antibodies did not bind to the endothelium from lung sections of injected mice. Immunohistochemistry assays of lung sections from mice injected with PBS (A and B) or GFP-Etx (C and D) were revealed with anti-mouse EnVision+ system-HRP (A and C) or anti-rabbit EnVision + system-HRP (B and D) as a secondary antibody. Incubations were developed as explained in the Materials and Methods sections but omitting the primary antibody. No binding was detected in the endothelium of any condition (arrowheads), with only a little background of some blood cells from lungs sections being revealed with the secondary antibody (brown, A and C). Nuclei were stained with hematoxylin. Scale bar 25 µm. [file 13567_2020_748_MOESM7_ESM.pdf]

**CONTROL**

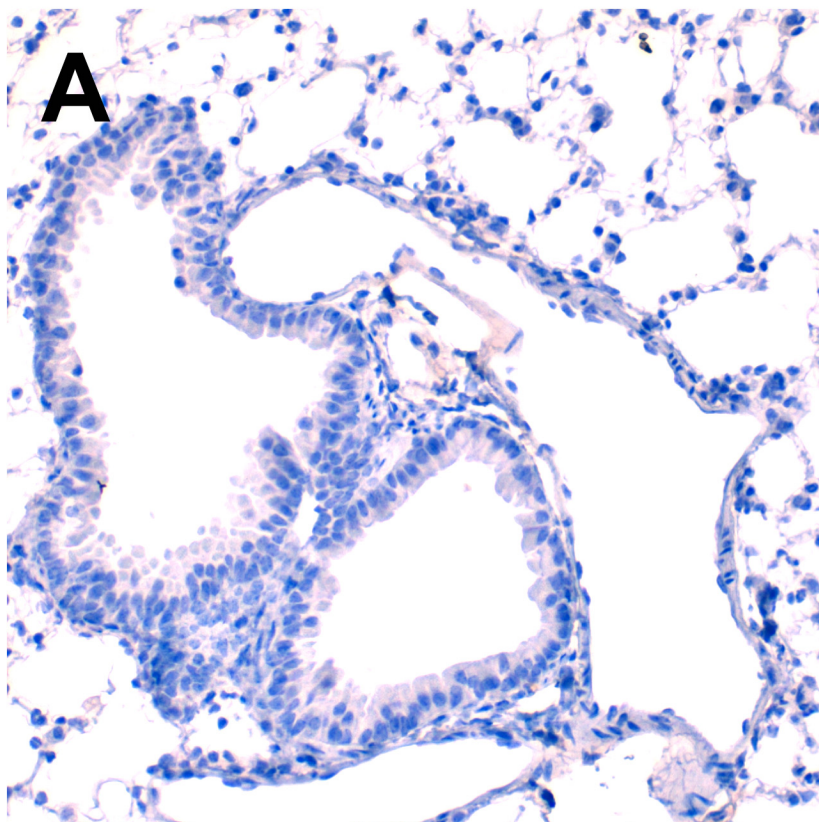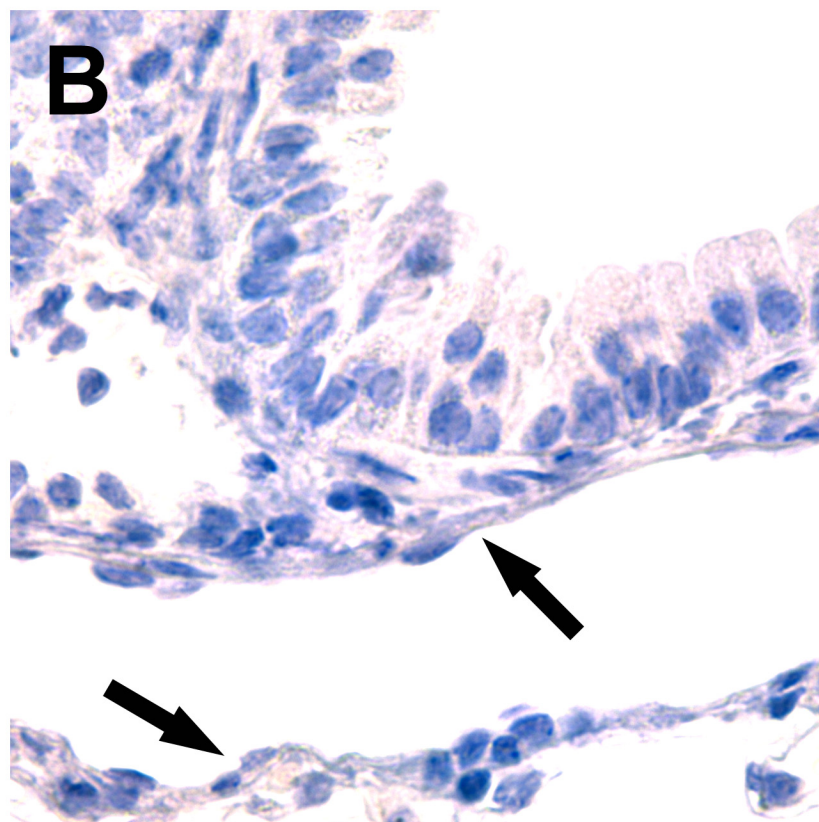

**$\alpha$ -MAL**

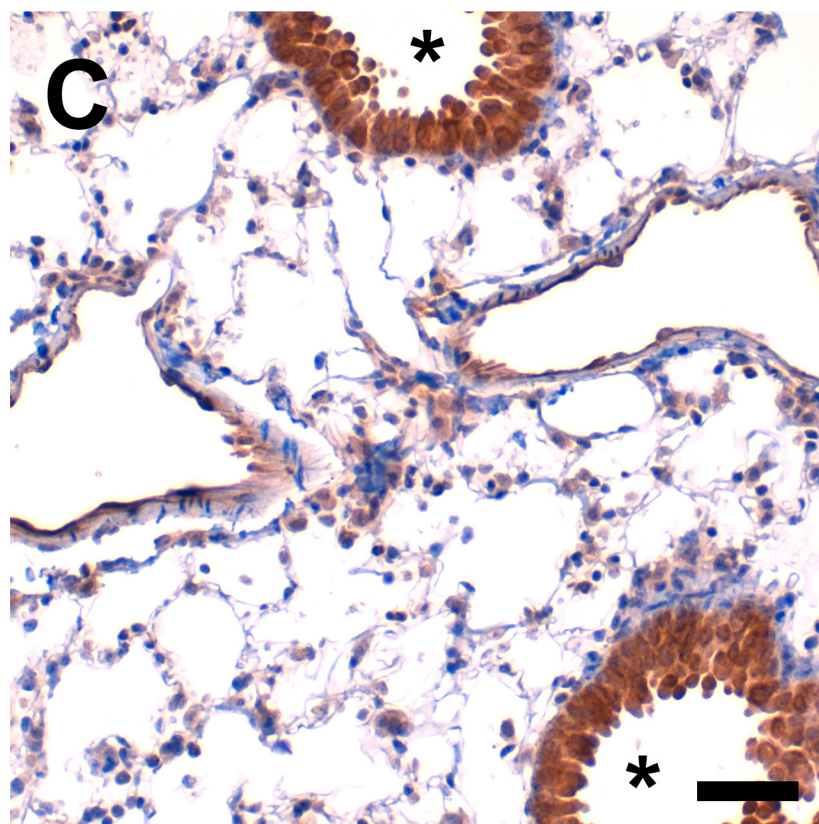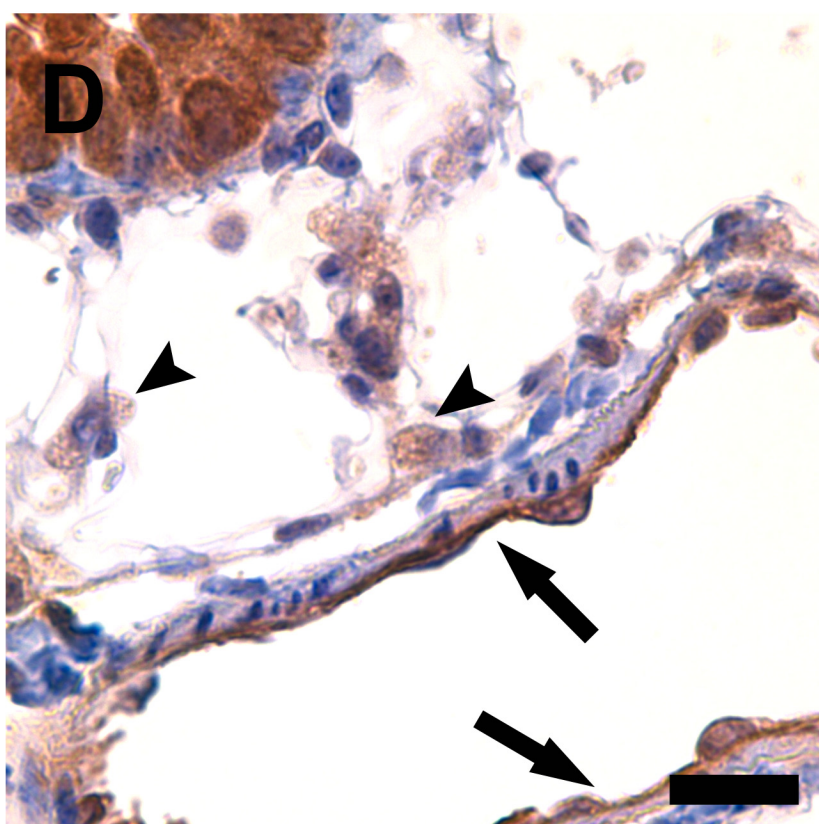

Supplement: Supplementary file 8 — Additional file 8. Anti-MAL staining on mouse lungs. Immunohistochemistry assays on lung sections from perfused mouse revealed MAL protein expression in endothelial cells of some vessels. Lung sections were incubated with the MAL antibody (C and D) or by omitting the primary antibody (A and B). All the sections were incubated with anti-mouse EnVision+ system-HRP and developed as explained in the Materials and Methods section. Note MAL protein expression in the endothelium (brown, arrows in D). However, it was not detected in the control conditions omitting the incubation with the primary antibody (arrows in B). MAL was also expressed in bronchial epithelial cells (brown, asterisk in C) and in type 2 pneumocytes (brown, arrowhead in D). B and D are magnifications from A and C images, respectively. Scale bars correspond to 50 µm in A and C, and 20 µm in B and D. [file 13567_2020_748_MOESM8_ESM.pdf]
